# Synthesis, Characterization, and Antimicrobial Activity of a Novel Trisazo Dye from 3-Amino-4H-thieno[3,4-c][1]benzopyran-4-one

**DOI:** 10.1155/2018/9197821

**Published:** 2018-02-01

**Authors:** Joseph Tsemeugne, Emmanuel Sopbué Fondjo, Jean-de-Dieu Tamokou, Taoufik Rohand, Arnaud Djintchui Ngongang, Jules Roger Kuiate, Beibam Luc Sondengam

**Affiliations:** ^1^Laboratory of Applied Synthetic Organic Chemistry, Department of Chemistry, Faculty of Science, University of Dschang, P.O. Box 67, Dschang, Cameroon; ^2^Department of Organic Chemistry, University of Yaoundé I, P.O. Box 812, Yaoundé, Cameroon; ^3^Laboratory of Microbiology and Antimicrobial Substances, Department of Biochemistry, Faculty of Science, University of Dschang, P.O. Box 067, Dschang, Cameroon; ^4^Laboratory of Analytical and Molecular Chemistry, Polydisciplinary Faculty of Safi, Cadi Ayyad University, Route Sidi Bouzid, BP 4162, 46000 Marrakech-Safi, Morocco

## Abstract

A new trisazo dye has been synthesized by coupling the diazonium ion of 3-amino-4H thieno[3,4-c][1]benzopyran-4-one with 2-*tert*-butyl-4-methoxyphenol. The newly prepared trisazo dye was characterized by its physical, elemental, and spectroscopic data. 2D-NMR (COSY, HSQC, and HMBC) techniques were used to secure the structural assignments. The new trisazo dye (compound** 7**) along with precursors** 3**,** 4**, and** 6** was screened by microdilution susceptibility assay for antibacterial and antifungal activities towards eight bacterial strains and three yeasts selected on the basis of their relevance as human pathogens. The results showed that compound** 7** (MIC = 2–128 *μ*g/mL) was the most active as compared with its precursors. The most resistant microorganisms were* V. cholerae *NB2 and* V. cholerae *SG24, whereas the most sensitive microorganism was* C. neoformans.* The overall results of this study indicated that compound** 7** had the greatest potential value against both yeasts and multidrug-resistant bacteria, so further investigation is warranted.

## 1. Introduction

The emergence of multidrug microbial resistance and the global warming are among the main challenges that the modern scientists have so far been facing in the recent decades. The fact that many pathogenic microorganisms responsible for several human and animal diseases have developed resistance mechanisms to the classical therapies has stimulated intensive investigations in the fields of natural and synthetic chemistry, with the aim of discovering new drug classes having much better therapeutic profiles. In this respect, the antimicrobial activities of numerous classes of organic substances such as phenols [1–3], coumarins [4–6], thiophenes [7–9], quinones [10, 11], and azo compounds [12–14] are currently the targets of the worldwide drug discovery efforts.

Interestingly, we are now seeing the emergence of a new generation of drugs with hybrid molecular architectures combining the biological features of two or more small molecules or even small molecules and biologics. The expectation is that, in the long term, such molecular and/or biologic conjugates could become a dominant form of targeted infectious diseases' therapies.

The idea of combining two or more potentially bioactive substructures to make an integrated new molecular framework with a higher anticipated therapeutic firepower is conceptually simple yet highly successful in the context of infectious diseases that require effective killing of the microbes.

The less use of azo compounds in medicinal chemistry is due to the fact that, in the presence of the enzymes of the organism, these compounds are readily reduced to aromatic amines which are reported to be highly mutagenic and carcinogenic [15], justifying therefore their marginal antimicrobial activities [16]. Nevertheless, it has been found that increasing the number of this group in the same compound results in the gradual inhibition of the biodegradation process. Hence, because of the low biodegradation rate of the polyazo compounds due to the important number of the –N=N– fragments in their structures, they should be preferred to their mono and disazo counterparts in the inhibition of the main pathogenic microorganisms of humans [17]. Moreover, previous studies have established the cumulative and synergetic effects of this functional group in the improvement of the biological activities of several hybrid lead compounds [18].

In this work, we report for the first time the synthesis of a new trisazo dye from 3-amino-4H-thieno[3,4-c][1]benzopyran-4-one and 2-*tert*-butyl-4-methoxyphenol, for further investigations in drug development against multidrug-resistant bacteria and fungi. We carried out the antimicrobial screenings of the new compound on eight bacterial strains (among which* V. cholerae *NB2,* V. cholerae *SG24,* V. cholerae CO6*, and* S. flexneri* are well known resistant strains) and three pathogen yeasts.

## 2. Experimental

### 2.1. Materials and Methods

#### 2.1.1. General Information

Melting point is corrected and was determined with an Electrothermal Melting Point Apparatus, model 9100. The TLC was carried out on Eastman Chromatogram Silica Gel Sheets (13181; 6060) with a fluorescent indicator. A mixture of ethyl acetate and methylene chloride (7 : 3) was used as an eluent and iodine was used as a revelator for the chromatograms. The IR spectra were measured with a Fourier Transform Infrared spectrometer JASCO FT/IR-4100. The UV spectrum was recorded with a Beckman DU-640 Spectrophotometer. Combustion analyses were carried out with a C, H, N, and S Euro EA from HEKAtech company, and results were found to be in good agreement (±0.3%) with the calculated values. EIMS were measured on Mass Spectrometer LCQ Classic with ESI Source from Thermo Fisher Scientific company. ^1^H-NMR spectrum was recorded in DMSO-*d*_6_ with a 250 MHz spectrometer Bruker AV III. ^13^C-NMR spectrum was recorded in DMSO-*d*_6_ with a 62.5 MHz spectrometer Bruker AV III. TMS was used as an internal reference.

#### 2.1.2. Preparation of the Reagents and Starting Materials

All the reagents mentioned in this work were purchased from Aldrich and Fluka and were used without further purification.

Starting material** 4** was prepared according to the procedures mentioned in the literature published earlier [19]. A mixture of 4.65 g of 4-methyl-2-oxo-2H-[1]benzopyran-3-carbonitrile (**3**) and sulphur (1.22 g, in excess) in ethanol (30 ml) was stirred using a magnetic plate shaker thermostated at 45°C. Ammonia (6 ml) was added dropwise during the first 10 min of the reaction. After 7 h of reaction, the resulting precipitate (5.08 g, 93%) was collected by filtration, washed with distilled water, and recrystallised in benzene to yield a yellow powder. m.p. 195-196°C (Lit. [19], 198-199°C, from benzene). IR (KBr, cm^−1^) *υ*_max_: 3449 (–NH_2_); 1687 (C=O); 1603 and 1547 (C=C); 1224 (C–O) cm^−1^. ^1^H NMR (250 MHz, DMSO-*d*_6_, Me_4_Si,*δ*, ppm): 7.87 (dd, 1H,* J* = 8.0 and 1.5 Hz, H-8), 7.78 (br s, 2H, NH_2_); 7.36 (ddd, 1H,* J* = 8.0, 7.4 and 1.8 Hz, H-6), 7.22–7.17 (m, 2H, H-7 and H-5), 6.89 (s, 1H, thiophenic H). ^13^C NMR (62.5 MHz, DMSO-*d*_6_, Me_4_Si,*δ*, ppm): 166.70 (C-3), 158.98 (C-2), 151 (C-4a), 130.90 (C-8a), 129.26 (C-9), 124.39 (C-6), 123.91 (C-8), 118.05 (C-2a), 117.09 (C-7), 98.07 (C-8b), 97.54 (C-5); *m*/*z* (EI) 217 (M+; 100%). Elemental Analysis: C_11_H_7_NO_2_S. Calculated: C: 60.83; H, 3.23; N, 6.45; S, 14.75. Found: C, 61.17; H, 3.36; N, 6.49; S, 14.45.

#### 2.1.3. Preparation of Diazonium Salt Solution

In a similar manner as described in [20], dry sodium nitrite (2.07 g, 3 mmol) was slowly added over a period of 30 minutes to concentrated sulphuric acid (10 mL) with occasional stirring. The solution was cooled to 0–5°C. Compound** 4** was dissolved in DMSO (10 mL) and cooled to 0–5°C. The nitrosylsulfuric acid solution was added to the solution of** 4** and the temperature was maintained between 0 and 5°C. The clear diazonium salt solution thus obtained consisting of the in situ formed intermediate** 5** was used immediately in the coupling reaction.

#### 2.1.4. 3-(2-{3-(*tert*-Butyl)-2-hydroxy-5-methoxy-4,6-bis[2-(4-oxo-4H-thieno[3,4-c]chromen-3-yl)diazenyl]pheny*l*}diazenyl)-4H-thieno[3,4-c]chromen-4-one Pentahydrate (**7**)

The phenol derivative 2-*tert*-butyl-4-methoxyphenol (540 mg; 3 mmol) was dissolved in DMSO (10 mL) and then cooled in an ice bath at 0–5°C. The diazonium solution of** 4** previously prepared was added dropwise over 1 hour, and then 15 mL of sodium acetate solution (10%) was added to the mixture. The pH of the mixtures was in the range 9–11. The solid precipitate was collected on a filter and crystallised from methanol to give the title** 7** (632 mg; 49%) as a green powder. m.p. 200°C. IR (KBr, cm^−1^) *υ*_max_: 3260 (OH), 3073 (Ar. C–H), 1721 (C=O), 1677 (Ar C=C), 1483, 1435 (N=N) cm^−1^. UV (THF) *λ*_max_/nm (log *ε*): 215 (2.95), 240 (4.56), 268 (4.18), 325.5 (4.74), 381 (4.48). ^1^H NMR (250 MHz, DMSO-*d*_6_, Me_4_Si,*δ*, ppm): 9.18 (d, 1H,* J* = 9.75 Hz, 9-H), 9.01 (d, 1H,* J* = 10.5 Hz, 9′′-H), 8.83 (d, 1H,* J* = 10.5 Hz, 9′′′-H), 8.59 (dd, 1H,* J* = 9.0 and 7.8 Hz, 8′′-H), 8.46 (dd, 1H,* J* = 10.5 and 9.75 Hz, 8′′′-H), 8.32 (dd, 1H,* J* = 9.75 and 9.75 Hz, 8-H), 8.27 (dd, 1H,* J* = 9.75 and 9.75 Hz, 7′′-H), 8.15 (dd, 1H,* J* = 9.75 and 9.0 Hz, 7′′′-H), 8.06 (dd, 1H,* J* = 11.25 and 9.0 Hz, 7-H), 7.99 (d, 1H,* J* = 9.0 Hz, 6-H), 7.97 (d, 1H,* J* = 9.6 Hz, 6′′-H), 7.96 (d, 1H,* J* = 7.0 Hz, 6′′′-H), 7.67 (s, 1H, 1-H), 7.58 (s, 1H, 1′′′-H), 7.50 (s, 1H, 1′′-H), 3.92 (s, 10H, H_2_O), 3.90 (s, 1H, OH), 3.50 (s, 3H, OCH_3_), 1.54 (s, 9H, CH_3_).^ 13^C NMR (62.5 MHz, DMSO-*d*_6_, Me_4_Si,*δ*, ppm): 164.9 (C-4), 164.2 (C-4′′ and C-4′′′), 157.3 (C-9b′′ and C-9b′′′), 157.2 (C-9b), 155.4 (C-5a′′), 155.3 (C-5a′′′), 154.5 (C-5a), 154.3 (C-3a′′), 154.0 (C-3a′′′), 153.2 (C-3a), 148.3 (C-2′), 138.2 (C-5′), 136.0 (C-3), 135.8 (C-3′′′), 134.9 (C-3′′), 131.6 (C-9a), 130.5 (C-9a′′), 129.7 (C-9a′′′), 129.6 (C-7), 129.4 (C-7′′), 129.1 (C-7′′′), 127.6 (C-9′′ and C-9′′′), 126.8 (C-9), 126.0 (C-8), 125.9 (C-8′′′), 125.8 (C-8′′), 120.5 (C-6′), 118.6 (C-4′), 117.7 (C-6), 117.5 (C-6′′), 117.6 (C-6′′′), 115.4 (C-1′′′), 115.3 (C-1′′), 114.7 (C-1), 113.8 (C-1′), 101.8 (C-3′), 56.7 (OCH_3_), 25.5 (C(CH_3_)_3_), 18.7 (3CH_3_). *m*/*z* (EI): 957(1) [M^+*∙*^ + 2H], 525 (1), 516 (2), 459 (21), 393 (3), 338 (5), 279 (1), 247 (19), 230 (6). Elemental Analysis: C_44_H_38_N_6_O_13_S_3_ Calculated: C, 55.34; H, 4.01; N, 8.80; S, 10.07. Found: C, 55.36; H, 3.99; N, 8.78; S, 9.99.

### 2.2. Antimicrobial Assay

#### 2.2.1. Microorganisms

A total of eight bacteria and three yeasts were tested for their susceptibility to the studied compounds. The microorganisms used in this study were taken from our laboratory collection. Among the clinical strains of* Vibrio cholerae *used in this study, strains NB2 and SG24 (1) belonged to O1 and O139 serotypes, respectively. These strains were able to produce cholera toxin and hemolysin [21, 22]. The other microbial species used in this study were* Candida parapsilosis* ATCC22019,* Candida albicans* ATCC9002,* Cryptococcus neoformans *IP95026,* Bacillus subtilis*,* Escherichia coli *S2 (1),* Vibrio cholerae *non-O1 and non-O139 (strains CO6 and PC2), and* Shigella flexneri*. The* V. cholerae *non-O1 and non-O139 strains were positive for hemolysin production but negative for cholera toxin production [22]. The bacterial and fungal species were grown at 37°C and maintained on nutrient agar (NA, Conda, Madrid, Spain) and Sabouraud Dextrose Agar (SDA, Conda) slants, respectively.

#### 2.2.2. Determination of Minimum Inhibitory Concentration (MIC) and Minimum Microbicidal Concentration (MMC)

The antimicrobial activity was investigated by determining the minimum inhibitory concentrations (MICs), minimum bactericidal concentrations (MBCs), and minimum fungicidal concentrations (MFCs). MIC of compounds was assessed using the broth microdilution method [23]. Each test compound was dissolved in dimethylsulfoxide (DMSO, Fisher Chemicals) to give a stock solution. This was serially diluted twofold in Mueller-Hinton Broth (MHB) for bacteria and Sabouraud Dextrose Broth (SDB) for fungi to obtain a concentration range of 512 to 0.25 *μ*g/ml. One hundred microliters of each concentration was introduced into a well (96-well microplate) containing 90 *μ*l of SDB or MHB, and 10 *μ*l of inoculum (1 × 10^6^ CFU/mL for bacteria and 1 × 10^5^ spores/ml for yeasts) was added to obtain a final concentration range of 256 to 0.125 *μ*g/ml. Plates were covered and incubated on the shaker at 37°C for 24 h (bacteria), 48 h (*Candida* spp.), and 72 h* (Cryptococcus)*. MICs were assessed visually after the corresponding incubation period and were taken as the lowest sample concentration at which there was no growth or virtually no growth.

For the minimum microbicidal concentration (MMC) determination, 10 *μ*l aliquots from each well that showed no growth of microorganism were plated on Mueller-Hinton Agar or Sabouraud Dextrose Agar and incubated at 37°C for 24 h (bacteria), 48 h (*Candida* spp.), and 72 h* (Cryptococcus)*. The lowest concentration that yielded no growth after the subculturing was taken as the MBCs or MFCs. Ciprofloxacin (Sigma-Aldrich, Steinheim, Germany) for bacteria and nystatin (Sigma-Aldrich, Steinheim, Germany) for yeasts were used as positive controls, while broth with 20 *μ*L of DMSO was used as a negative control. The assay was repeated thrice.

## 3. Results and Discussion

### 3.1. Chemistry

**Scheme 1:**
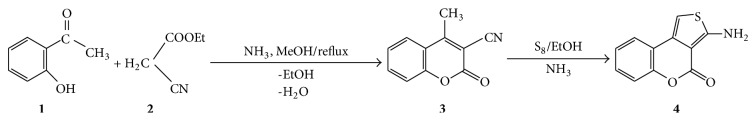
Reaction sequences to compound** 4**.

The title 2-aminothiophene reagent** 4** was prepared in excellent yield by applying the third version of the Gewald methodology (Scheme 1) as previously reported [19, 24–26].

**Scheme 2:**
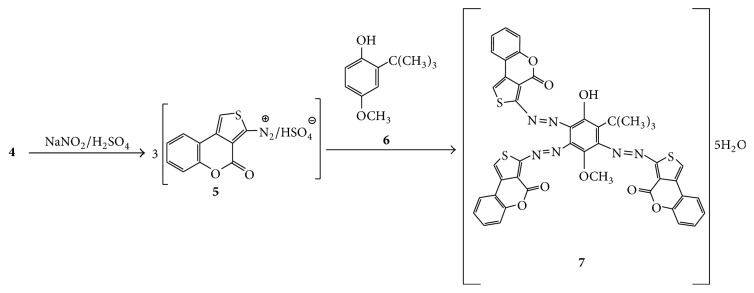
Reaction sequences to compound** 7**.

The in situ formed intermediate thienyl diazonium sulphate** 5** (Scheme 2) was generated from the diazotization of compound** 4** using nitrosylsulfuric acid at a very low temperature (0–5°C). The freshly prepared diazonium solution was then coupled with phenolic reagent 2-*tert*-butyl-4-methoxyphenol** 6**. The resulting mixture was worked up as usual to yield the azo compound** 7** (Scheme 2).

The structure of the newly synthesized compound** 7** was elucidated by IR, UV, NMR, mass spectral studies, and elemental analysis.

The IR spectrum of compound** 7** showed absorption bands ranging between 3260 and 2324 cm^−1^ due to the stretching vibration of OH of phenol and H_2_O molecules. Around 1721 cm^−1^ appeared a band of stretching frequencies of the carbonyl functionality, whereas the characteristic stretching frequencies of the –N=N– linkages were exhibited around 1435 cm^−1^. The absorption bands at 1677 cm^−1^ and 763 cm^−1^ depicted the presence of C=C and Ar–H, respectively. The infrared spectra also showed a distinctive band at 1051 cm^−1^ due to C–O bond of the methoxy group.

The electronic transitions of the UV-visible spectra in DMSO give rise to wavelength *λ*_max_ ranging from 215 to 381 nm as a result of *π* → *π* ^*∗*^ transitions of the compound indicating the presence of an extended *π*-conjugation of the C=C backbone enhanced by the N=N bridges linking the three thienocoumarins moieties to the parent phenolic benzene ring. Five strong absorption bands were observed, whereby those appearing at *λ*_max_ 215 nm (log *ε* = 2.95) and *λ*_max_ 240 nm (log *ε* = 4.56) may be attributed to *π* → *π* ^*∗*^ transition of the benzenoid moiety of the compounds and intraligand *π* → *π* ^*∗*^ transition. The third and fourth bands located at *λ*_max_ 268 nm (log *ε* = 4.18) and *λ*_max_ 325.5 nm (log *ε* = 4.74) were attributed to the electronic *n* → *π* ^*∗*^ transitions of the –N=N– group. The last band appearing in the visible region around *λ*_max_ 381 nm (log *ε* = 4.48) is probably related to a transition involving the whole electronic system of the azo dyes.

**Scheme 3:**
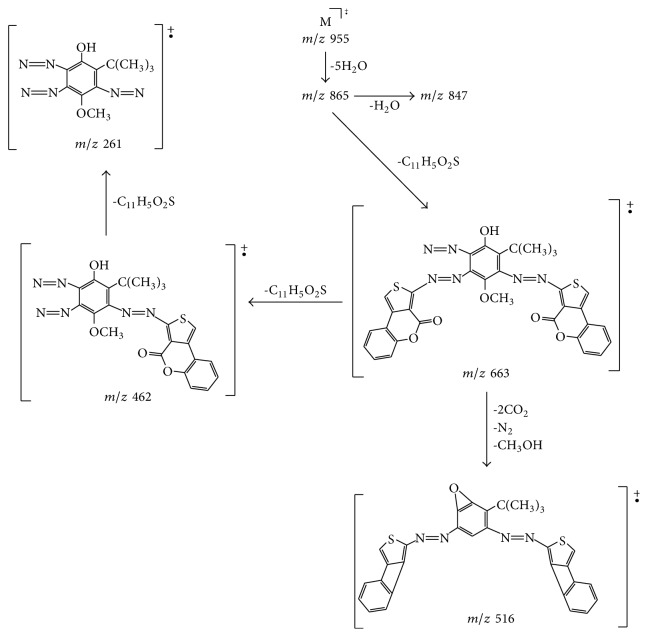
Significant HRMS fragmentation patterns of compound** 7**.

The structure of compound** 7** is further strongly supported by its elemental analysis and its HREIMS, which showed a molecular ion peak at *m*/*z* 955. The mass spectrum of compound** 7** exhibited fragment ions at *m*/*z* 910, 865, and 847 which could be assigned to [M^+*∙*^  − COOH], [M^+*∙*^ − 5H_2_O], and [M^+*∙*^  − 6H_2_O], respectively. The ion fragments at *m*/*z* = 663, 616, and 230 were assigned as in Scheme 3, confirming the above structural hypothesis.

On the ^1^H-NMR spectrum, the singlets observed at *δ*_H_ = 3.50 and 1.54 ppm were, respectively, assigned to the protons of the methoxy group and the protons of the 3 methyl substituents of the* tert*-butyl group. The thiophenic protons resonated at *δ*_H_ = 7.67, 7.50, and 7.58 ppm as a singlet, while the singlets observed at *δ*_H_ = 9.04 ppm were assigned to the OH group. The ^1^H-NMR also showed a set of three couples of poorly resolved “ddd”-signals [centered at 8.06 (H-7,* J* = 11.25 and 9.0 Hz), 8.32 (H-8,* J* = 9.75 and 9.75 Hz), 8.27 (H-7′′,* J* = 9.75 and 9.75 Hz), 8.59 (H-8′′,* J* = 9.0 and 7.8 Hz), 8.15 (H-7′′′,* J* = 9.75 and 9.0 Hz), and 8.46 (H-8′′′,* J* = 10.5 and 9.75 Hz), ppm] and a set of three couples of poorly resolved “dd”-signals [centered at 7.99 (H-6,* J* = 9.0 Hz), 9.18 (H-9,* J* = 9.75 Hz), 7.97 (H-6′′,* J* = 9.6 Hz), 9.01 (H-9′′,* J* = 10.5 Hz), 7.96 (H-6′′′,* J* = 7.0 Hz), and 8.83 (H-9′′′,* J* = 10.5 Hz)] overlapping in the range 7.97–9.18 ppm, which were attributed to the protons of the three thienocoumarin substructural units of the coupling product. The ^1^H-^1^H-COSY experiment was advantageously used in the accurate assignments of the different ^1^H-^1^H connectivities [27].

**Figure 1:**
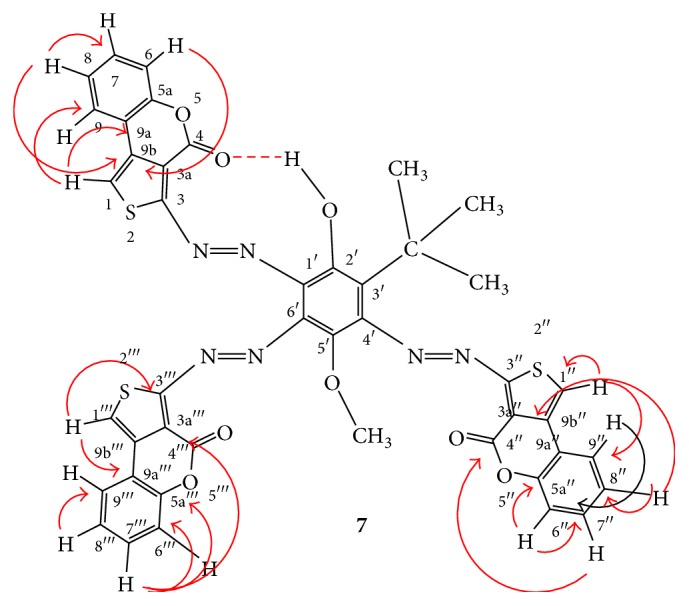
HMBC interactions in compound 7.

**Figure 2:**
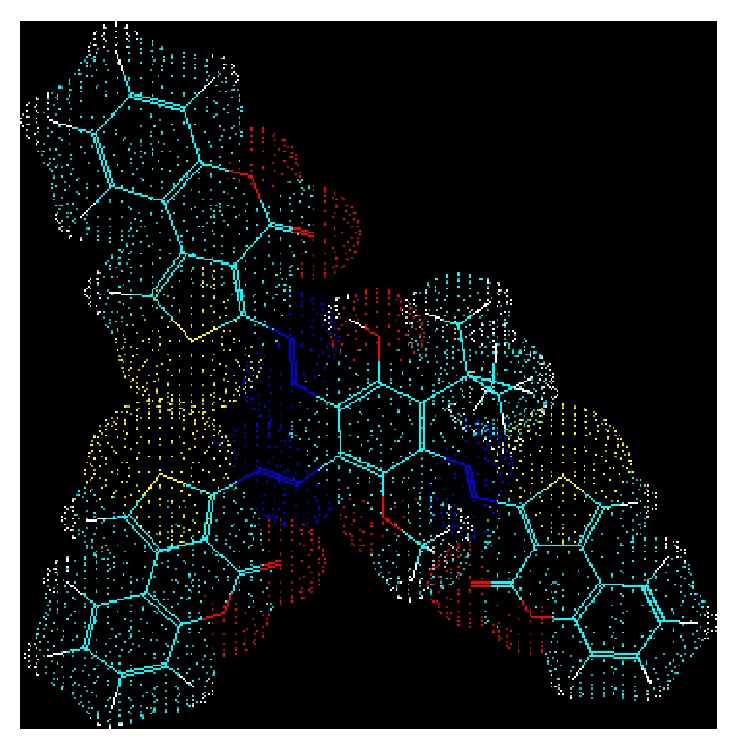
Optimized 3D view of compound** 7**. The structure was drawn with the program ACD/3D viewer (freeware) of ACD/Labs.

The ^13^C-NMR spectrum of compound** 7** displayed forty carbon signals instead of forty-four as required by the molecular formula. This could be explained by the overlapping of the signals of the three methyl substituents of the* tert*-butyl group at *δ*_C_ 18.72 ppm due to their magnetic and chemical equivalence. Furthermore, it was assumed that the signals of two of the three carbonyl functionalities overlapped to give the intense signal at *δ*_C_ 164.17 ppm besides the signal at *δ*_C_ 164.94 ppm assigned to the other carbonyl group. A close inspection of the 3D view (Figure 2) of the optimized structure geometry of compound** 7** clearly reveals that the electron clouds of the carbonyl groups of the two 3-diazenyl-4H-thieno[3,4-c]chromen-4-one moieties attached at positions −3 and −5 of the central phenolic ring are virtually more involved in the interactions with the electron clouds of the neighboring atoms and groups than the carbonyl group of the 3-diazenyl-4H-thieno[3,4-c]chromen-4-one moiety fixed at position −2 of the core phenolic ring. These observations could justify the slight upfield shifts (Δ*δ* = 166.70–164.17 = 2.53 ppm for the two overlapping carbonyl groups and Δ*δ* = 166.70–164.94 = 1.76 ppm for the other one) that occurred in the resonances of the three carbonyl functions when compared to the chemical shift (*δ*_C_ = 166.70 ppm) of the corresponding carbonyl group in the starting compound** 4**. Based on the DEPT-90/135 experiments, the remaining signals were assigned without ambiguity to the aromatic C atoms bearing hydrogen atoms. The HMBC experiments [27] were used to disclose the two-bond (^2^ *J*_CH_), three-bond (^3^ *J*_CH_), and occasionally four-bond (^4^ *J*_CH_) couplings (Table 1 and Figure 1) between the hydrogen atoms and the tertiary and quaternary carbon atoms, thereby facilitating their identification and their assignments.

### 3.2. Antimicrobial Activity

The antimicrobial activity of new trisazo dye along with some of its precursors was examined by microdilution susceptibility assay against eight bacterial strains and three yeasts selected on the basis of their relevance as human pathogens. The results showed that the tested compounds exhibited variable MICs (2 to 256 *μ*g/mL) depending on the microbial strains (Table 2). The most resistant microorganisms were* V. cholerae *NB2 and* V. cholerae *SG24, whereas the most sensitive microorganism was* C. neoformans. *Compound** 7** (MIC = 2–128 *μ*g/mL) was more active as compared with its precursors followed in a decreasing order by compound** 4** (MIC = 4–256 *μ*g/mL), compound** 6** (MIC = 8–>256 *μ*g/mL), and compound** 3** (MIC = 32–>256 *μ*g/mL).

The lowest MIC value of 2 *μ*g/mL was recorded on* S. aureus *with compound** 7**, whereas the lowest MMC value of 4 *μ*g/mL was obtained on* C. parapsilosis* and* C. neoformans* with compound** 7**. However, the highest MIC value of 256 *μ*g/mL was recorded on* V. cholerae *CO6 with compound** 3** and on* V. cholerae* NB2 with compounds** 4** and** 6**, while the highest MMC value of 256 *μ*g/mL was obtained on* V. cholerae* SG24 with compound** 7** and on* C. parapsilosis*,* S. flexneri*,* E. coli*, and* B. subtilis *with compound** 3**. A lower MBC/MIC (≤4) value signifies that a minimum amount of compounds is used to kill the microbial species, whereas a higher value signifies the use of comparatively higher amounts of samples for the control of any microorganism [28].

The strains of* V. cholerae* NB2 and PC2 [21, 22] and* Shigella flexneri* [29] included in the present study were MDR clinical isolates and these were resistant to commonly used drugs such as ampicillin, streptomycin, tetracycline, nalidixic acid, furazolidone, and cotrimoxazole. However, these bacterial strains were found to be sensitive to most of the tested samples, suggesting that their administration may represent an alternative treatment against the diseases caused by these types of MDR pathogenic strains.

From the SAR point of view, we can say that compounds** 4** and** 6** are not microbicides for the bacterial strains* V. cholerae NB2*,* S. flexneri*, and* V. cholerae SG24*, with respect to the MBC/MIC data displayed in Table 2. In contrast, compound** 7 **is microbicidal for these bacterial strains. The improved antibacterial profiles of the hybrid compound could be due to the synergetic interactions of the primary potent pharmacophores combined in the single molecular platform. The cumulative effects of the three disazo scaffolds certainly contributed to a greater extent to shaping the global biological profile of the new hybrid molecule. These observations that corroborate recent findings from analogous investigation [18].

## 4. Conclusion

In summary, the diazotized title 2-aminothiophene derivative** 5** is a powerful electrophilic reagent which attacks phenolic substrate** 6** at all its three nonsubstituted ring positions to give with good yield the corresponding trisazo hybrid compound** 7** whose structural assignment was done on the basis of the available analytic and spectroscopic data. The combination in a single molecular platform of the pharmacophores related to the phenolic, the three thienocoumarin, and the three disazo moieties resulted in a synergy of actions which globally contributed as initially anticipated to enhancing the antimicrobial activities of the reaction product** 7** when compared with the profiles of the precursors** 3**,** 4**, and** 6**. The results showed that the diazotization followed by the coupling reaction increases the antimicrobial activity of the trisazo compound compared with that of the free 2-aminothiophene and phenol. The overall results of this study indicated that compound** 7** had the greatest potential value against both yeasts and multidrug-resistant bacteria, so further investigation is warranted.

## Figures and Tables

**Table 1:** Important HMBC interactions in compound **7**; ^1^H and ^13^C chemical shifts (*δ*/ppm) in DMSO-*d*_6_ as the solvent (25°C).

Compound **7**			Compound **7**		
C atom	*δ* ^13^C	HMBC (H → C)	C atom	*δ* ^13^C	HMBC (H → C)
7	129.6	H-8 (8.32)	9a′′	130.5	H-8′′ (8.59), H-7′′ (8.27), H-6′′ (7.97)
9	126.8	H-1 (7.67)	1′′′	115.4	H-9′′′ (8.83), H-1′′′ (7.58)
9a	131.6	H-1 (7.67)	3′′′	135.8	H-9′′′ (8.83), H-7′′′ (8.15), H-1′′′ (7.58)
9b	157.2	H-8 (8.32), H-6 (7.99)	4′′′	164.2	H-7′′′ (8.15)
1′′	115.3	H-1′′ (7.50)	5a′′′	155.3	H-9′′′ (8.83), H-7′′′ (8.15)
5a′′	155.4	H-6′′ (7.97)	6′′′	117.6	H-7′′′ (8.15)
7′′	129.4	H-9′′ (9.01), H-6′′ (7.97)	9′′′	127.6	H-8′′′ (8.46), H-1′′′ (7.58)
8′′	125.8	H-8′′ (8.59)	9a′′′	129.7	H-1′′′ (7.58)
9′′	127.6	H-1′′ (7.50)			

**Table 2:** Minimum inhibitory concentrations (MICs) and minimum microbicidal concentrations (MMCs) (*µ*g/ml) of compound **7** and its entire precursors against fungal and bacterial strains.

Microorganisms	Inhibition parameters	**3**	**4**	**6**	**7**	Reference drugs^*∗*^
*Gram-positive bacteria*						
*Bacillus subtilis *	MIC	64	16	64	8	2
	MBC	256	32	>256	32	2
*S. aureus*	MIC	64	16	32	2	0.5
	MBC	>256	32	64	8	0.5
*Gram-negative bacteria*						
*E. coli *S2(1)	MIC	64	32	32	8	2
	MBC	256	64	>256	32	2
*S. flexneri *	MIC	128	32	128	16	8
	MBC	256	>256	>256	32	8
*P. aeruginosa *	MIC	128	16	128	8	4
	MBC	>256	32	>256	32	4
*V. cholerae *NB2	MIC	>256	256	256	64	32
	MBC	nd	>256	>256	128	32
*V. cholerae *SG24	MIC	>256	>256	>256	128	64
	MBC	nd	nd	nd	256	64
*V. cholerae CO6*	MIC	256	64	>256	32	8
	MBC	>256	128	nd	64	8

*Yeasts *						
*C. parapsilosis* ATCC22019	MIC	64	4	32	4	2
	MFC	256	8	64	4	2
*C. albicans* ATCC9002	MIC	64	8	64	4	4
	MFC	>256	16	128	8	4
*C. neoformans *IP95026	MIC	32	4	8	4	2
	MFC	128	8	16	4	2

^*∗*^Ciprofloxacin for bacteria and nystatin for yeasts; nd: not determined; compounds **1**, **2**, and **5** were not tested.

## Supplementary Materials

Supplementary Materials Figure  1: MS spectrum of compound 4. Figure  2: 1H-NMR spectra of compound 4 (DMSO-d6, 250 MHz). Figure  3: 13C-NMR spectrum of compound 4 (DMSO-d6, 62.5 MHz). Figure  4: UV-visible spectrum of compound 7. Figure  5: IR spectrum of compound 7. Figure  6: full 1H-NMR spectrum of compound 7 (DMSO-d6, 250 MHz). Figure  7: expanded 1H-NMR spectrum of compound 7 (DMSO-d6, 250 MHz). Figure  8: 13C-NMR spectrum (aromatic region) of compound 7 (DMSO-d6, 62.5 MHz). Figure  9: full 13C-NMR spectrum of compound 7 (DMSO-d6, 62.5 MHz). Figure  10: HMBC spectrum of compound 7 (DMSO-d6, 62.5 MHz). Figure  11: MS spectrum of compound 7.
