# Supplementary material for: Synthesis, Characterization, and Antimicrobial Activity of a Novel Trisazo Dye from 3-Amino-4H-thieno[3,4-c][1]benzopyran-4-one
Source: Int J Med Chem. 2018 Feb 1;2018:9197821. doi: 10.1155/2018/9197821 (PMC5816859; doi:10.1155/2018/9197821)
Supplement: Supplementary Materials — Figure 1: MS spectrum of compound 4. Figure 2: 1H-NMR spectra of compound 4 (DMSO-d6, 250 MHz). Figure 3: 13C-NMR spectrum of compound 4 (DMSO-d6, 62.5 MHz). Figure 4: UV-visible spectrum of compound 7. Figure 5: IR spectrum of compound 7. Figure 6: full 1H-NMR spectrum of compound 7 (DMSO-d6, 250 MHz). Figure 7: expanded 1H-NMR spectrum of compound 7 (DMSO-d6, 250 MHz). Figure 8: 13C-NMR spectrum (aromatic region) of compound 7 (DMSO-d6, 62.5 MHz). Figure 9: full 13C-NMR spectrum of compound 7 (DMSO-d6, 62.5 MHz). Figure 10: HMBC spectrum of compound 7 (DMSO-d6, 62.5 MHz). Figure 11: MS spectrum of compound 7. [file 9197821.f1.pdf]

SUPPLEMENTARY MATERIALS

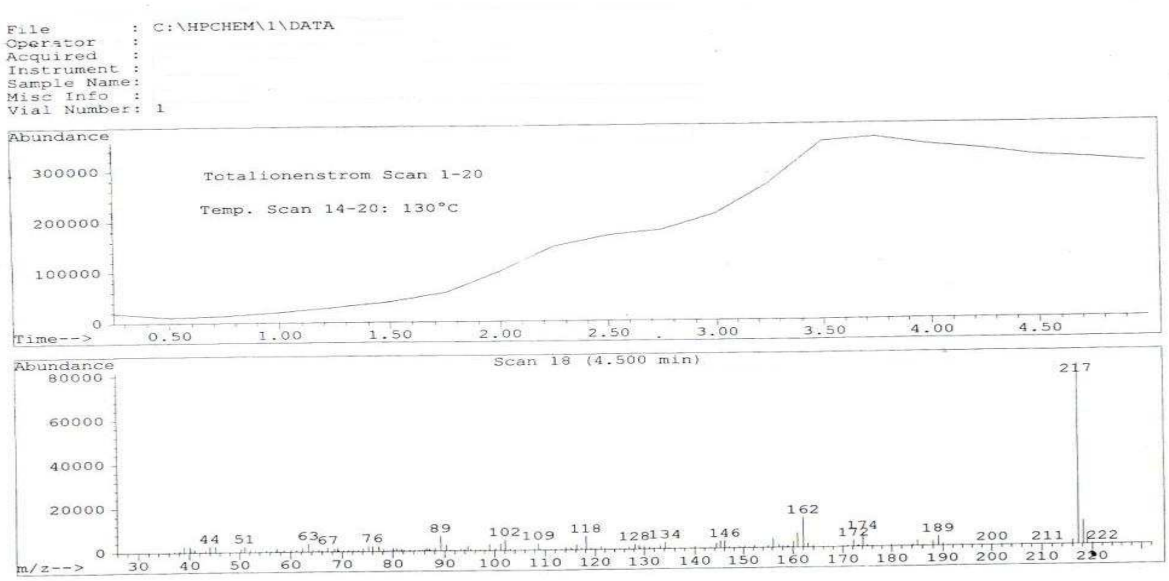

MS Spectrum of Compound 4

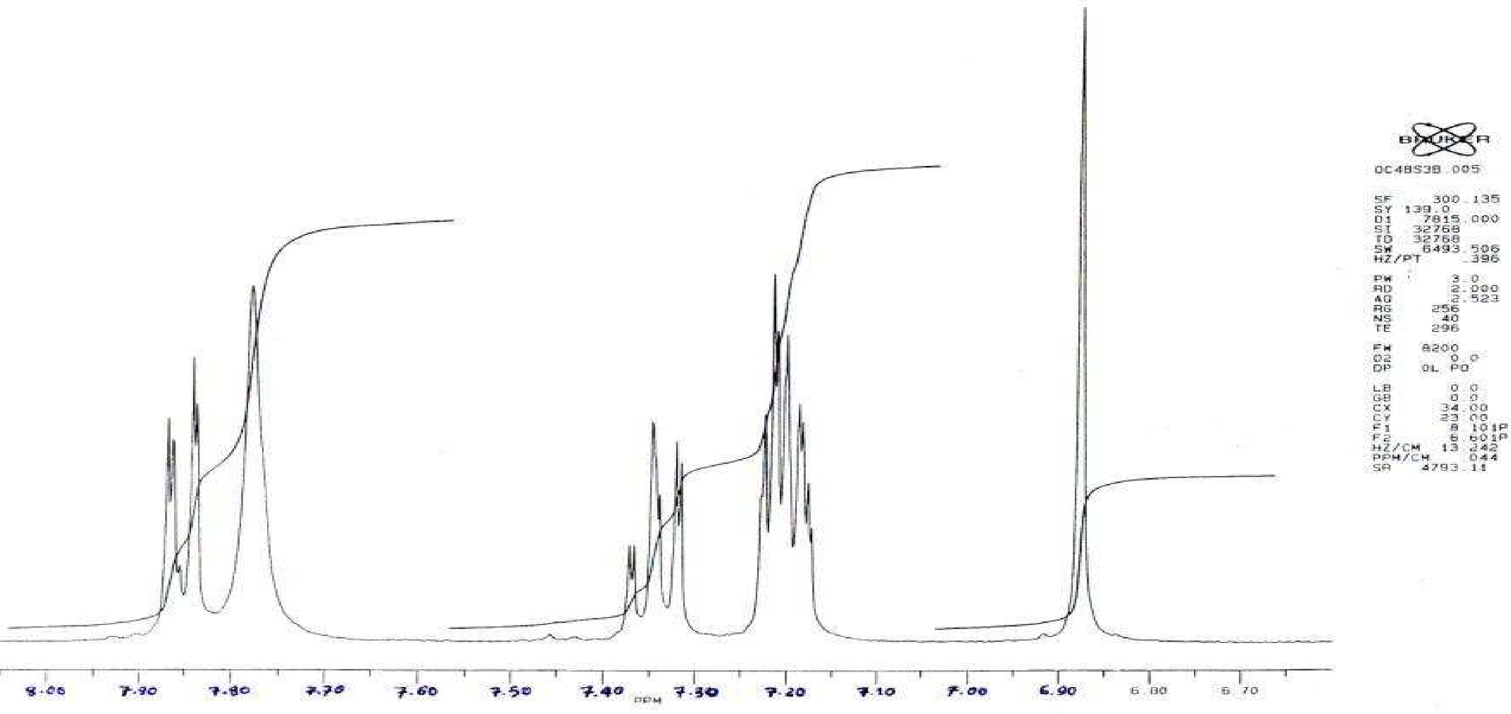

^{1}H-NMR Spectra of compound 4 (DMSO-d_6, 250 MHz)

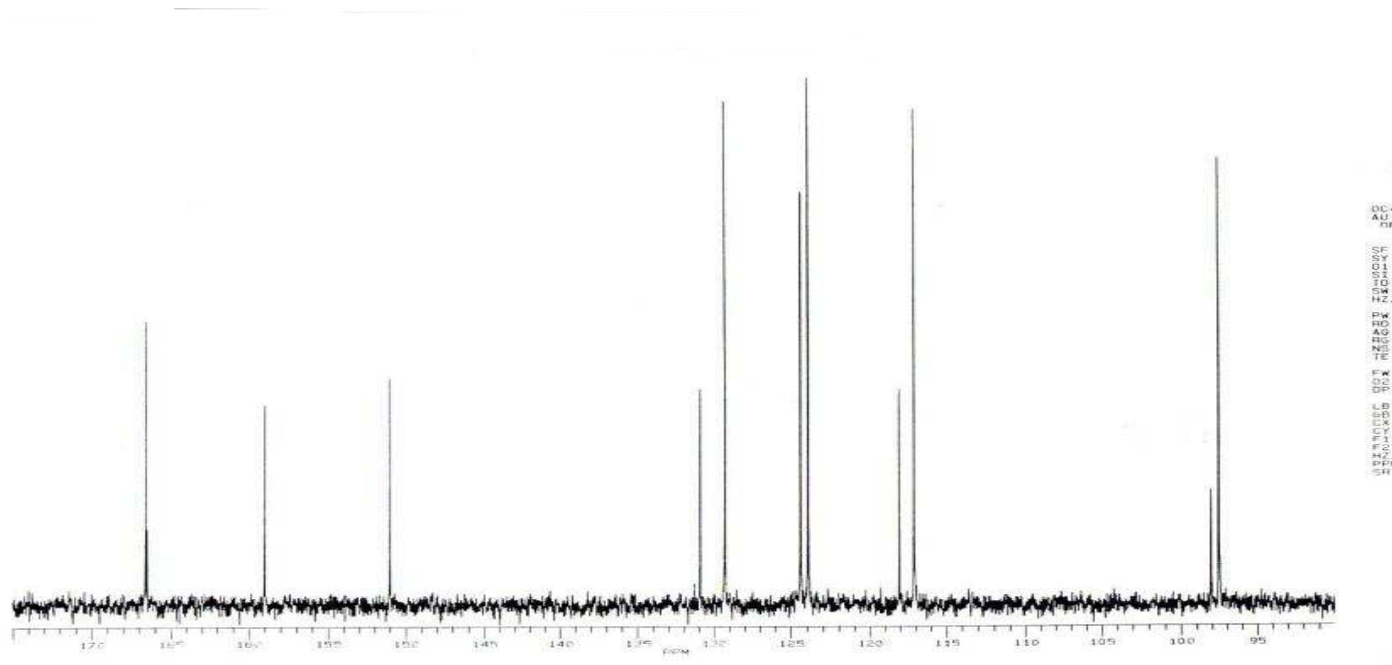

^{13}C-NMR Spectrum compound 4 (DMSO-d_6, 62.5 MHz)

<u>UV-VISIBLE spectrum of compound 7</u>

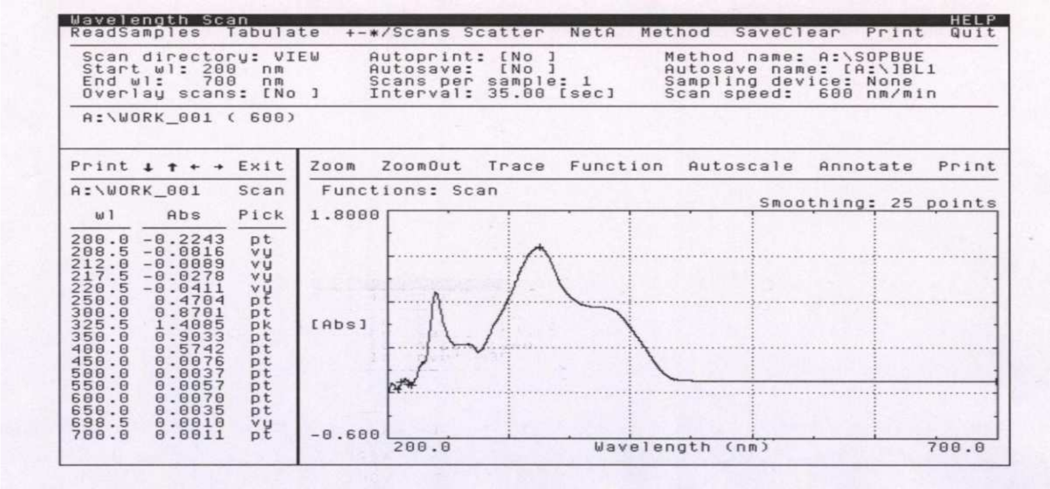

<u>IR spectrum of compound 7</u>

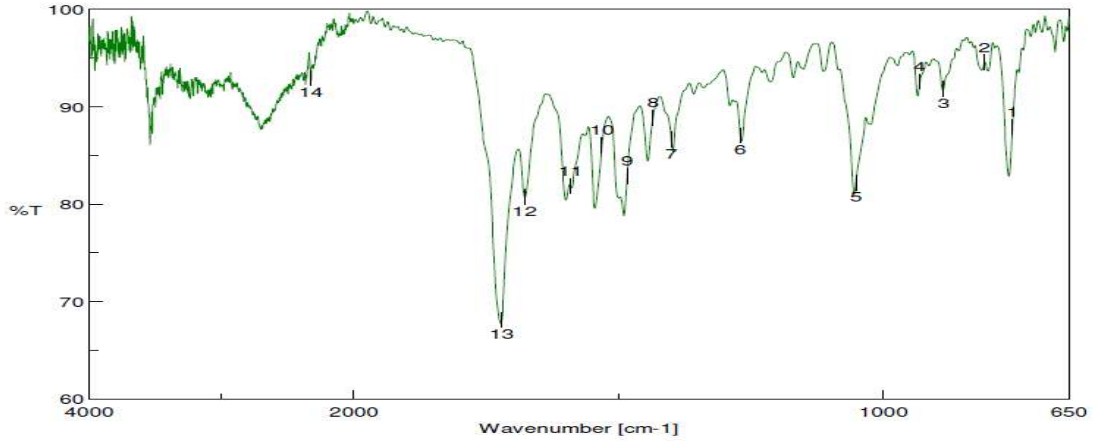

Normal ^{1}H- and ^{13}C-NMR, ^{1}H, ^{1}H-COSY and HMBC spectra of compound 62

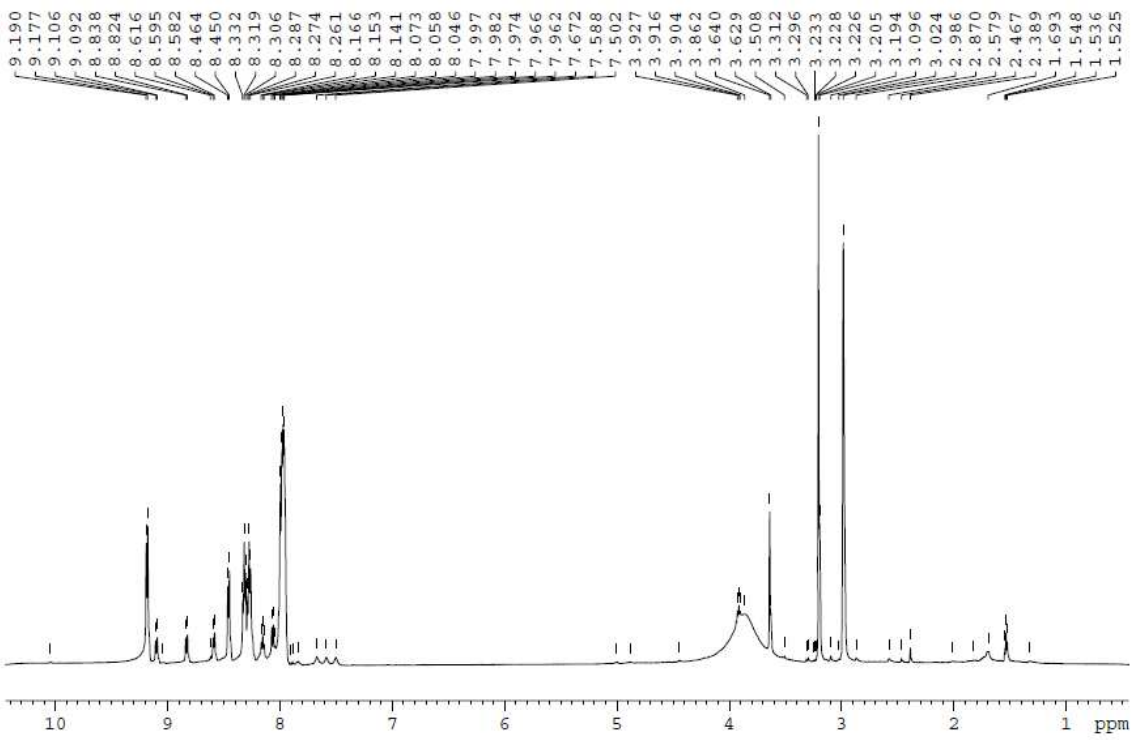

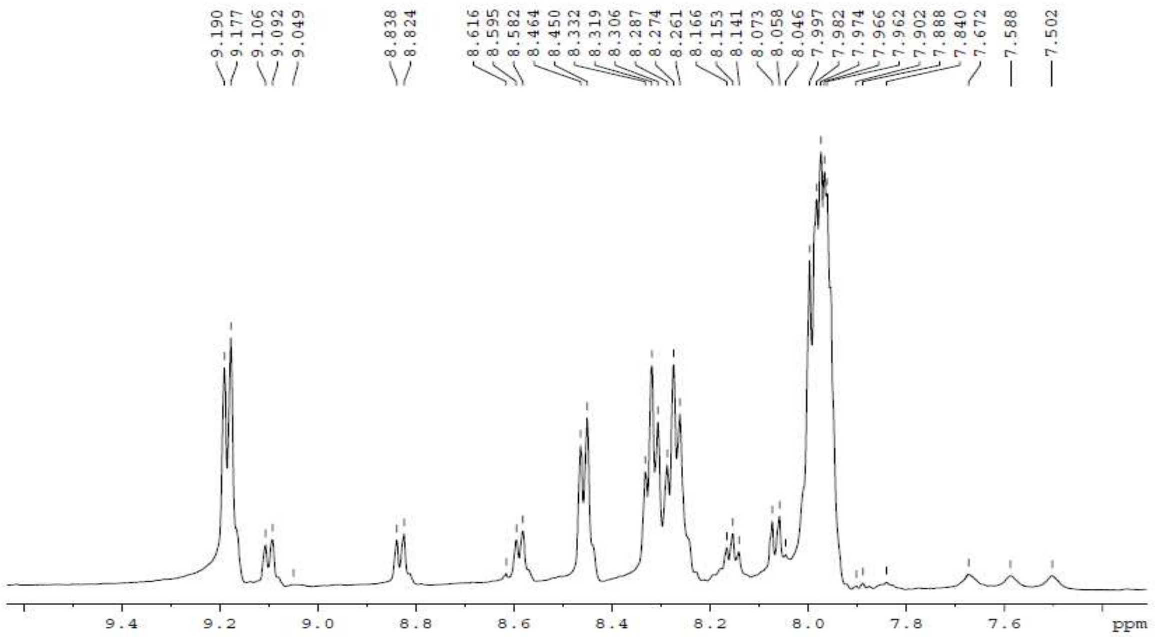

^{1}H-NMR Spectra of compound 7 (DMSO-d_6, 250 MHz)

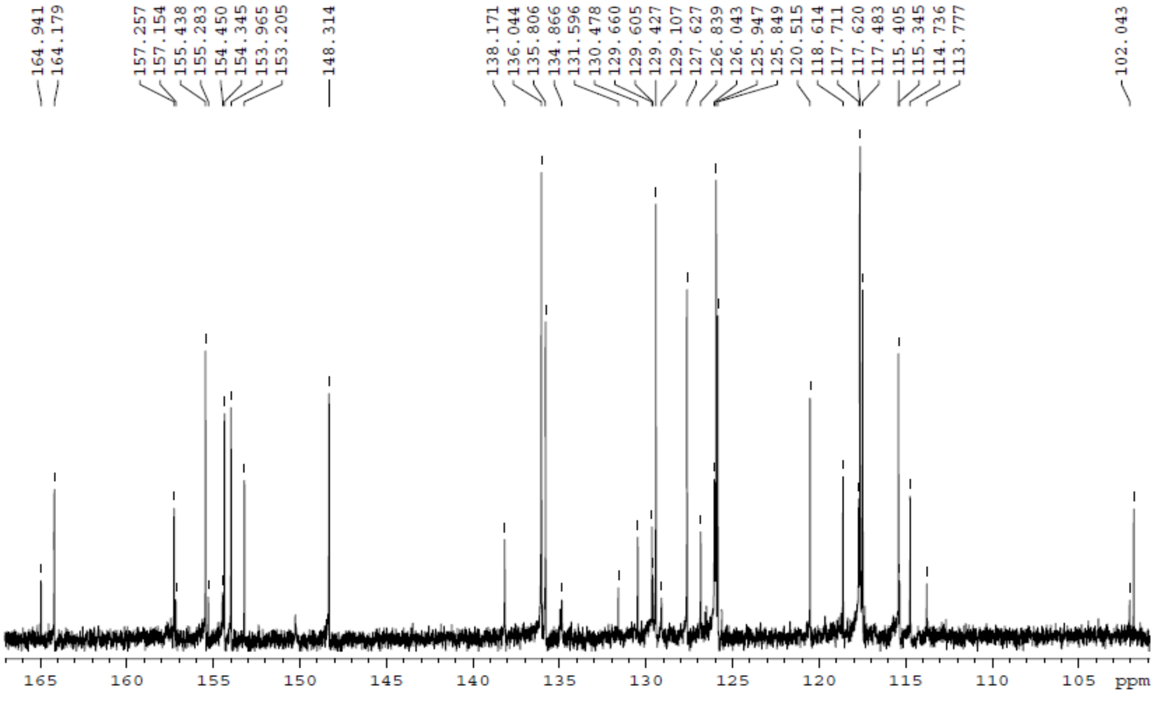

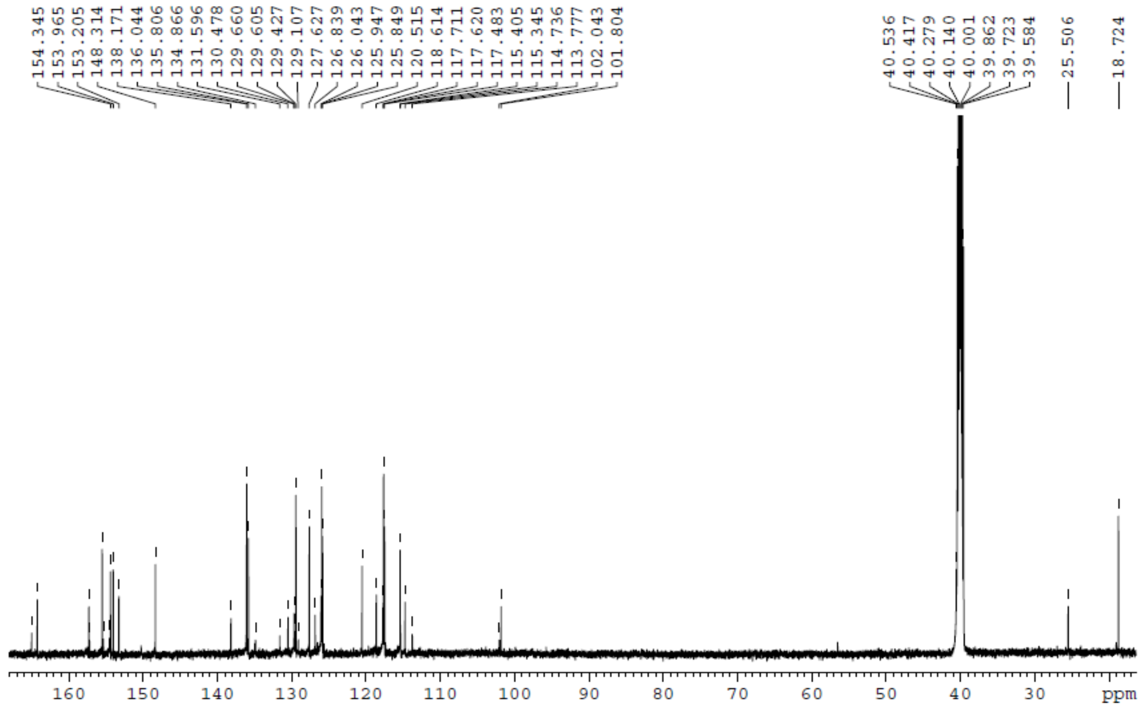

^{13}C-NMR Spectrum compound 7 (DMSO-d_6, 62.5 MHz)

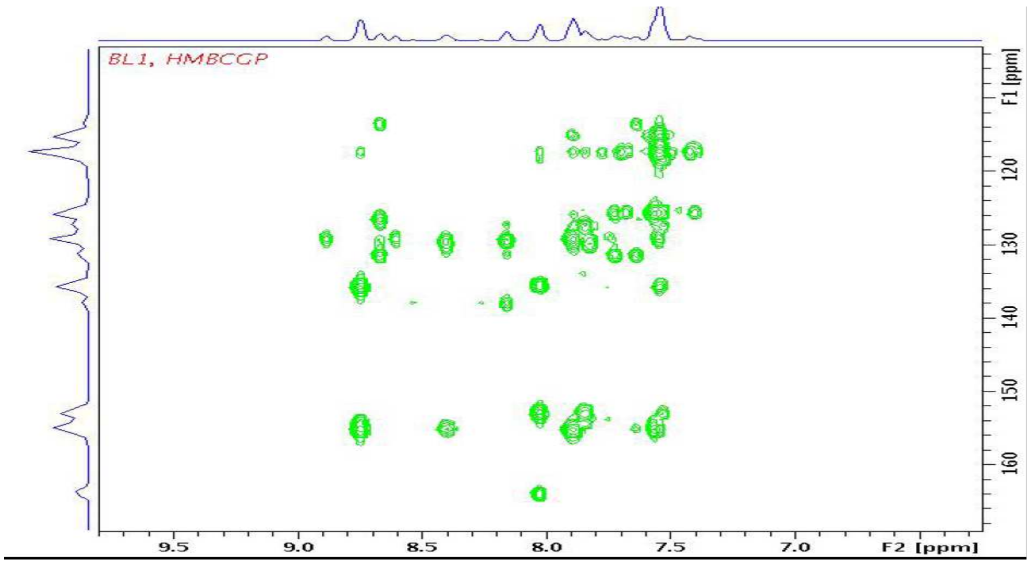

HMBC Spectrum of Compound 7 (DMSO-d_6, 62.5 MHz)

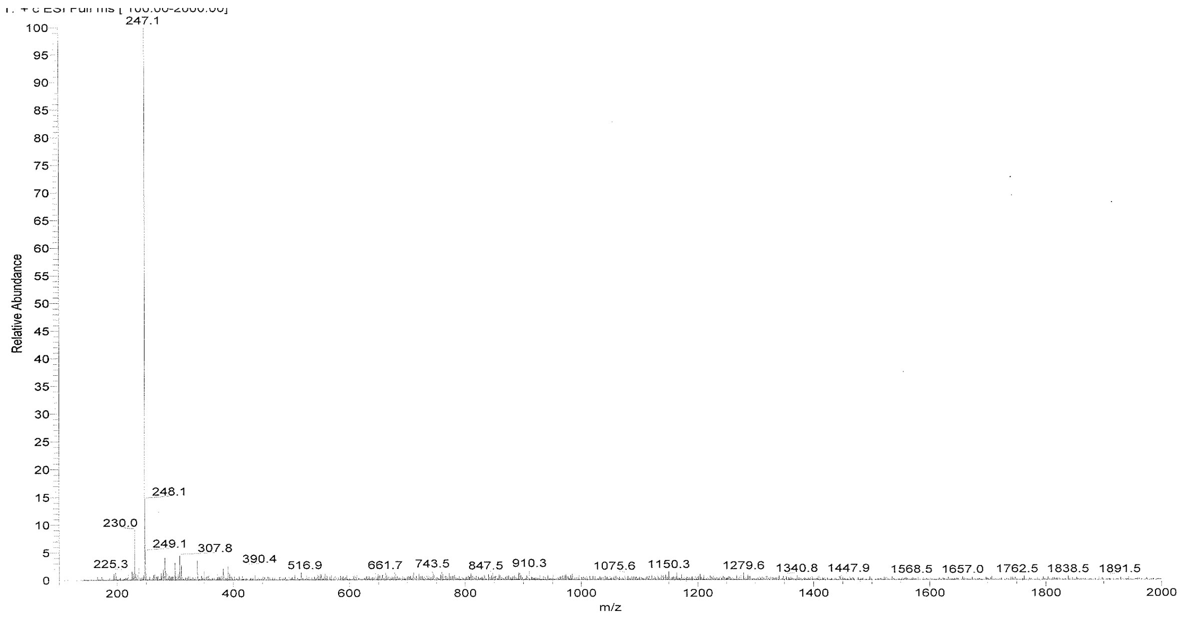

MS Spectrum of Compound 7
